# Uric Acid and Xanthine Levels in Pregnancy Complicated by Gestational Diabetes Mellitus—The Effect on Adverse Pregnancy Outcomes

**DOI:** 10.3390/ijms19113696

**Published:** 2018-11-21

**Authors:** Anna Pleskacova, Vendula Bartakova, Katarina Chalasova, Lukas Pacal, Katerina Kankova, Josef Tomandl

**Affiliations:** 1 Department of Pathophysiology, Faculty of Medicine, Masaryk University, 625 00 Brno, Czech Republic; pleskacova@med.muni.cz (A.P.); vbartak@med.muni.cz (V.B.); katarina.kuricova@gmail.com (K.C.); paci@med.muni.cz (L.P.); kankov@med.muni.cz (K.K.); 2 Department of Biochemistry, Faculty of Medicine, Masaryk University, 625 00 Brno, Czech Republic

**Keywords:** uric acid, uricemia, xanthine, gestational diabetes mellitus, pregnancy, adverse perinatal outcomes

## Abstract

Uric acid (UA) levels are associated with many diseases including those related to lifestyle. The aim of this study was to evaluate the influence of clinical and anthropometric parameters on UA and xanthine (X) levels during pregnancy and postpartum in women with physiological pregnancy and pregnancy complicated by gestational diabetes mellitus (GDM), and to evaluate their impact on adverse perinatal outcomes. A total of 143 participants were included. Analyte levels were determined by HPLC with ultraviolet detection (HPLC-UV). Several single-nucleotide polymorphisms (SNPs) in UA transporters were genotyped using commercial assays. UA levels were higher within GDM women with pre-gestational obesity, those in high-risk groups, and those who required insulin during pregnancy. X levels were higher in the GDM group during pregnancy and also postpartum. Positive correlations between UA and X levels with body mass index (BMI) and glycemia levels were found. Gestational age at delivery was negatively correlated with UA and X levels postpartum. Postpartum X levels were significantly higher in women who underwent caesarean sections. Our data support a possible link between increased UA levels and a high-risk GDM subtype. UA levels were higher among women whose glucose tolerance was severely disturbed. Mid-gestational UA and X levels were not linked to adverse perinatal outcomes.

## 1. Introduction

Uric acid (UA) is a terminal product of purine metabolism with non-negligible extracellular antioxidant function; however, increased UA levels are established markers of pathological mechanisms involved in a plethora of diseases [1]. UA production is maintained by an enzyme, xanthine oxidoreductase (XOR), which catalyzes the oxidation from hypoxanthine to xanthine (X) and the subsequent oxidation from X to UA [2]. Long-term hyperuricemia (i.e., levels higher than 360 μmol/L in women and 420 μmol/L in men) typically seen in gout or even short-term elevation associated with tumor lysis syndrome may lead to the formation of urate calculi in kidney and other tissues. Despite its limited solubility and a fate of waste product, UA is largely (~90%) reabsorbed in the kidney and the intestine and returned to the blood. Increased UA levels may occur as a result of an increased UA production, impaired renal UA excretion, or both [3]. Hyperuricemia itself is linked to atherosclerosis and basically to all components of metabolic syndrome such as visceral obesity, hypertension, dyslipidemia, insulin resistance, and diabetes mellitus [4], and several studies showed UA to be one of the hallmarks of the metabolic syndrome [5,6,7,8].

Serum UA levels show substantial genetic components; 28 single-nucleotide polymorphisms (SNPs) affecting UA levels were identified so far using a genome-wide association approach [9]. Of those variations, those in the transporters *SLC2A9* and *ABCG2* seem to have a major effect. The gene *SLC2A9* encodes glucose transporter 9 (GLUT9) which is present in the basolateral membrane of proximal tubular cells and mediates release of urate reabsorbed from the primary filtrate. Genetic variation in the *SLC2A9* explains 3.5% of UA level variation [10] and is associated with both UA levels and gout. Interestingly, the effect of genetic variation was reported stronger in women [11]. ATP-binding cassette G2 (ABCG2) is a transport protein expressed in the apical membrane of proximal tubular cells where it contributes substantially to the urate efflux in the primary filtrate, as shown by a functional study. A common non-synonymous SNP rs2231142 in the *ABCG2* decreases urate efflux by 53% [12].

Insulin resistance increases during the second trimester of pregnancy and resolves upon delivery under physiological conditions. However, reduced β-cell reserve or their maladaptation to higher insulin demands may result in gestational diabetes mellitus (GDM) [13]. GDM is defined as diabetes first diagnosed in the second or third trimester of pregnancy that is not clearly either pre-existing type 1 or type 2 diabetes [14]. Pregnancies affected by GDM pose an increased risk of perinatal complications for both mother and child [15], such as macrosomia, lower gestational age at delivery, the need for induced labor or caesarean section, and further increased perinatal morbidity and mortality. Glucose homeostasis abnormality usually resolves soon after delivery; however, women with a history of previous GDM remain at an increased risk for recurrent GDM in subsequent pregnancies, as well as long-term risk of development of permanent type of diabetes and cardiovascular diseases [16,17]. Furthermore, children from pregnancies affected by GDM tend to have non-optimal body mass index (BMI) and impaired glucose tolerance or type 2 diabetes mellitus (T2DM) in early adulthood more often than those born to non-GDM mothers [18,19].

During physiological pregnancy, serum UA levels normally decrease until approximately the 24th week of gestation due to a substantial increase of glomerular filtration, decreased reabsorption in renal tubules, and the effect of estrogen; however, UA levels later return to normal around the term. UA transfers freely from maternal to fetal circulation, and negative correlations between offspring weight and UA levels were repeatedly described [20,21]. Rasika et al. [22] reported no significant correlation between UA levels in a period of 24–28th week of gestation and the risk of GDM development; however, early-pregnancy UA levels (before the 15th week of gestation) were significantly correlated with GDM risk, and UA levels before the 20th week of pregnancy were associated with GDM and mild preeclampsia [23]. Aker et al. [24] reported linear association of GDM or impaired glucose tolerance with first-trimester serum UA levels in Turkish women. Güngör et al. [25] described higher UA levels in GDM subjects compared to healthy controls in the 24th week of gestation; however, the difference was not statistically significant. Laughon et al. [26] associated increased UA levels with insulin resistance in mid-pregnancy, and hyperuricemia was associated with lower birth weight in the same study. Elevated UA levels were often associated with preeclampsia [27,28], and they were further shown to be a predictor for adverse maternal and perinatal outcomes in women with preeclampsia and hypertensive disorders [20,29,30]. However, a systematic review by Thangaratinam et al. [31] reported UA as a rather poor predictor. Amniotic fluid UA levels were shown to be a predictor of infant birth [32]; furthermore, higher UA levels were also associated with lower birth weight in normotensive women without insulin resistance (determined using a homeostatic model assessment for insulin resistance (HOMA-IR)) [33]. However, the evidence for the pathogenic relationship between gestational UA levels and child weight, preeclampsia, or hypertensive adverse pregnancy outcomes is scarce so far. 

In summary, there is strong evidence that hyperuricemia is linked to metabolic syndrome and T2DM in the general population. However, the available knowledge regarding UA levels during pregnancy complicated by GDM and during postpartum period in women with GDM history is controversial so far. Recently, Leng at al. [34] assessed the risks of postpartum T2DM and prediabetes development among Chinese women with previous GDM and found positive linear association of serum UA as a continuous variable with the risks of T2DM or prediabetes in cubic spline models and also increased multivariable-adjusted odds ratios for diabetes risk across quartiles of serum UA. Previously published studies on the relationship between UA and GDM did not take into account other metabolites in the UA pathway (such as X levels) as a possible marker of XOR activity; thus, our aims were (i) to determine mid-gestational and early postpartum UA and X levels and compare them between pregnant women with or without GDM, (ii) to analyze the relationship between mid-gestational and postpartum UA levels and parameters related to glucose intolerance (i.e., fasting plasma glucose (FPG) and oral glucose tolerance test (oGTT) values), (iii) to analyze the relationship between UA levels and selected markers of metabolic syndrome, (iv) to evaluate the effect of UA on perinatal outcomes, and (v) to study possible genetic contributions (i.e., genotype–phenotype relationships) to UA levels in pregnancy mediated by selected SNPs in genes encoding UA transporters (*ABCG2* rs2231142 and *SLC2A9* rs1014290, rs12498742, rs16890979, and rs734553).

## 2. Results

### 2.1. Study Subjects and Stratification Criteria

A total of 143 pregnant Caucasian women were enrolled in the study. Controls (*n* = 34) were women with physiological pregnancies who passed routine GDM screening in mid-trimester with negative results. GDM cases (*n* = 109) were women that fulfilled GDM criteria in mid-gestational screening and who also participated in postpartum oGTT (within the period of six weeks to 12 months after delivery). The persistent postpartum disturbance of glucose metabolism was ascertained in 5.9% of post-GDM women within the time frame up to one year after delivery. Clinical and anthropometric characteristics of both study groups are provided in Table 1.

Women with FPG ≥ 7.0 mmol/L in mid-gestational oGTT were considered as having diabetes with the first recognition in pregnancy (DIP) according to reference [35]. DIP was present in 5.5% of our GDM cases. For the purpose of more detailed analysis, we stratified GDM cases for the risk of adverse perinatal outcomes into (a) low-risk group (*n* = 56; 51.4% of all GDM cases) and (b) high-risk group (*n* = 53; 48.6%) using the following criteria: need for insulin to compensate GDM and/or abnormal fetal growth and/or pre-gestational BMI > 30 and/or increased weight gain during pregnancy > 20 kg and/or hypertension.

Perinatal characteristics (gestational age at delivery, spontaneous or induced labor, length of delivery, caesarean section, Apgar score, cord-blood pH, and base excess) and data on post-delivery complications (manual lysis, revision of the uterine cavity, and hypotonia) were available for 91 women who gave birth at the Department of Obstetrics and Gynecology in the University Hospital Brno (of those, 57 were GDM cases and 34 controls). Gestational age at delivery before the 37th week was considered a preterm birth. Protracted delivery was considered as delivery exceeding 480 min. Apgar score below 5 in the fifth minute after delivery, an umbilical cord-blood pH immediately after birth below 7.1, and base excess below −12 were considered pathological. 

### 2.2. Comparison of Biochemical and Anthropometrical Data

GDM women were significantly elder than women in the control group; however, there was no statistically significant difference in pre-gestational BMI (*p* = 0.040 and *p* = not significant (NS), respectively, Mann–Whitney). History of previous GDM and family history of diabetes mellitus (DM) was higher in GDM women (*p* = 0.009 and *p* < 10^−4^, Fisher exact test). In line with many previous reports, weight gain during pregnancy and also offspring birth weight were lower in the GDM group compared to the control group (*p* < 10^−6^ and *p* = 0.032, respectively, Mann–Whitney), most likely as a result of good compliance of women with newly diagnosed GDM following dietary recommendations (see Table 1). None of our study participants had clinical hyperuricemia either at mid-trimester (the threshold for UA is 262 μmol/L according to Abbassi-Ghanavati et al. [36]) or postpartum (the threshold is 360 μmol/L).

X levels during pregnancy were positively correlated with mid-gestational BMI, 1-h post-load plasma glucose (1-h PPG), and 2-h post load plasma glucose (2-h PPG) (*r* = 0.18, *p* = 0.037; *r* = 0.28, *p* = 0.033; and *r* = 0.23, *p* =0.0092, respectively, Spearman correlation coefficient). In both groups, X levels were higher postpartum compared to mid-gestation (both *p* < 0.001, Wilcoxon test). Furthermore, X levels were significantly higher in the GDM group compared to the control group during pregnancy, as well as postpartum (*p* = 0.049 and *p* = 0.0002, respectively, Mann–Whitney; see Table 1). However, the intrinsic difference (in the nanomolar range) was negligible and, therefore, biologically likely irrelevant despite being statistically significant. Similarly, UA levels were increased in both groups postpartum compared to their mid-pregnancy levels (both *p* < 10^−4^, Wilcoxon test); however, no statistically significant differences in UA levels were found between the GDM and control groups during pregnancy or postpartum (both *p* = NS, Mann–Whitney; see Table 1). UA levels in mid-pregnancy were positively correlated with pre-gestational BMI, FPG, 2-h PPG, and area under the curve (AUC) of oGTT in mid-trimester in group of GDM women (*r* = 0.23, *p* = 0.018; *r* = 0.36, *p* = 0.00012; *r* = 0.21, *p* = 0.029; and *r* = 0.35, *p* = 0.00022, respectively, Spearman correlation coefficient), but none of these correlations were significant in the control group (all *p* = NS, Spearman correlation coefficient). The oGTT in the period of six weeks to 12 months postpartum was available only for post-GDM women whose postpartum UA levels were positively correlated with FPG, 2-h PPG, and AUC of postpartum oGTT (*r* = 0.25, *p* = 0.012; *r* = 0.25, *p* = 0.011; and *r* = 0.31, *p* = 0.0016, respectively, Spearman correlation coefficient) and also with pre-gestational BMI (*r* = 0.27, *p* = 0.0041, Spearman correlation coefficient).

### 2.3. GDM Sub-Group Analysis

Given that the exact pathophysiological background of GDM is still unclear, and that UA levels exhibit high inter-individual variability, we performed in-depth analyses of UA levels on the stratified patient population, i.e., high vs. low risk of adverse perinatal outcomes GDM groups, and also considering the risk criteria/parameters individually. X levels were intrinsic again (in the nanomolar range) and, therefore, biologically likely irrelevant despite being statistically significant; as such, the results are not shown. Considering individual criteria, UA levels during pregnancy and also postpartum were significantly higher in GDM women with the occurrence of pre-gestational overweight or obesity (BMI ≥ 25) (*p* = 0.0088 and *p* = 0.041, respectively, Mann–Whitney). Moreover, when the sub-group of GDM treated by insulin was assessed separately, pre-gestational BMI and UA levels both during pregnancy and postpartum were higher (*p* = 0.039, *p* = 0.041, and *p* = 0.049, respectively, Mann–Whitney) in the sub-group requiring insulin to maintain optimal glucose levels compared to GDM women on diet only. Otherwise, no statistically significant differences between UA levels were found in the sub-group of GDM women with DIP and those who developed postpartum glucose metabolism disturbance within one year after delivery; however, both sub-groups were very small and, thus, both comparisons lack statistical power. Upon stratification of GDM women for increased risk of perinatal outcomes using the multiparametric risk classifier, the high-risk GDM group had higher mid-gestation UA levels (*p* = 0.02, Mann–Whitney), together with higher FPG, AUC of mid-gestational oGTT, and higher AUC of postpartum oGTT (*p* = 0.0022, *p* = 0.0067, and *p* = 0.049, respectively, Mann–Whitney).

### 2.4. Labor and Perinatal Outcome Data

Gestational age at delivery, occurrence of premature labor, and the incidence of spontaneous onset of labor, protracted delivery, post-delivery complications (manual lysis, revision of the uterine cavity, and hypotonia), pathological Apgar score, abnormalities of cord-blood pH, and base excess did not differ between women with and without GDM (all *p* = NS, Mann–Whitney or Fisher exact test; see Table 2). Solely gestational age at delivery was negatively correlated to UA and X levels postpartum (*r* = −0.22, *p* = 0.021 and *r* = −0.22, *p* =0.020, respectively, Spearman correlation coefficient), but none of the other listed outcomes were associated with either UA or X levels (all *p* = NS, Mann–Whitney). The caesarean section rate was significantly higher in a group of GDM women (*p* = 0.043, Fisher exact test; see Table 2), and postpartum X levels were significantly higher in a group of women who underwent caesarean section (*p* = 0.039, Mann–Whitney).

### 2.5. Analyses of Selected SNPs in UA Transporters

Genotype frequencies of four of a total of five studied SNPs were in Hardy–Weinberg equilibrium (*p* > 0.05, chi-square); rs12498742 deviated from Hardy–Weinberg equilibrium in the control group due to the small size of this group. Neither genotype nor allele distributions of the studied SNPs differed between GDM women and controls (all *p* = NS, chi-square; see Table 3).

**Figure 1:**
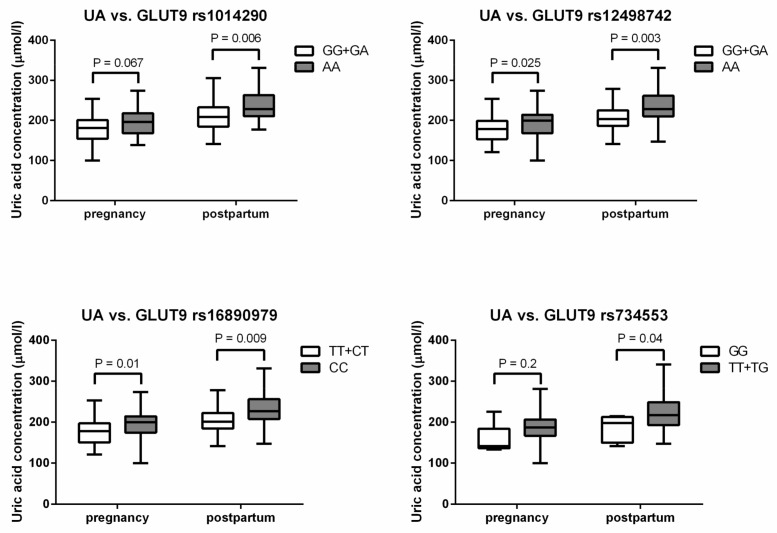
Uric acid (UA) levels according to single-nucleotide polymorphisms (SNPs) in glucose transporter 9 (GLUT9; *SLC2A9* gene). Box and whisker plots were constructed as medians, minimum and maximum values, and interquartile ranges. Statistics were calculated using Mann–Whitney test.

Furthermore, genotype–phenotype relationships were analyzed for all studied SNPs. UA levels differed significantly between carriers of genotypes of GLUT9 rs1014290 and rs12498742 postpartum, and rs734553 and rs16890979 both during pregnancy and postpartum (*p* = 0.0053, *p* = 0.0017, *p* = 0.024, *p* = 0.0043, *p* = 0.024, and *p* = 0.0043, respectively, Kruskal–Wallis ANOVA). Borderline significance was found for rs1014290 and rs12498742 in GLUT9 during pregnancy (*p* = 0.050 and *p* = 0.096, respectively, Kruskal–Wallis ANOVA). Allele G in rs1014290 and rs12498742, allele T in rs16890979, and homozygote GG in rs734553 in GLUT9 were associated with lower UA levels, as shown in Figure 1.

When we analyzed those genotypes that were correlated with increased levels of UA simultaneously (AA in rs1014290 and rs12498742, CC in rs16890979, and allele T in rs734553), we found increased levels of UA during pregnancy and also postpartum (*p* = 0.035 and *p* = 0.016, Mann–Whitney test). However, the presence of GDM cases did not differ between those genotype carriers (*p* = NS, Fisher exact test).

## 3. Discussion

Abnormalities of UA metabolism and handling are implicated in the pathogenesis of metabolic syndrome and diabetes. Since previous, albeit so far inconclusive, studies on UA metabolism in GDM indicated a possible link, we designed the current study with the aim of replicating eventual findings on another (central European in this case) population and to extend the research on other metabolites (xanthine) and putative genetic determinants. Here, we report the results of the study combining case-control and a follow-up approach including also selected offspring data to provide as complex as possible a picture of UA involvement in most common pregnancy complications and metabolic outcomes. Major findings of the current study can be summarized as follows: (i) UA levels were higher in women with pre-gestational overweight/obesity, in the high-risk group (as defined earlier), and in those who required insulin therapy; (ii) UA levels were positively correlated with BMI and glycemia both during pregnancy and postpartum; (iii) gestational age at delivery was negatively correlated with UA levels postpartum; and (iv) the genetic component for UA levels was confirmed for rs1014290, rs12498742, rs734553, and rs16890979 in GLUT9 (encoded by the *SLC2A9* gene).

Unlike in other species and most mammals, both UA and X are waste products in humans and higher primates. Other purines including hypoxanthine can be reincorporated into the cell pool and are salvaged from the blood [37]. UA also undergoes reabsorption in the kidney and serves several physiological purposes including antioxidant/radical-scavenging capacity, blood pressure regulation, or blood–brain barrier stabilization [1,38,39]. On the contrary, hyperuricemia was proposed as a significant contributor to pro-inflammatory endocrine imbalance in adipose tissue leading to a low-grade inflammation and insulin resistance present in metabolic syndrome [40], and the importance of UA metabolism in diabetes was shown as the inhibition of UA synthesis ameliorates metabolic syndrome and increases insulin sensitivity [41,42].

There are two possible mechanisms for hyperuricemia worsening hyperinsulinemia and aggravating glucose metabolism disturbances: (i) through UA-mediated inhibition of NO generation via several mechanisms [43] (insulin-stimulated release of NO from endothelia mediates glucose uptake into the skeletal muscle); and (ii) through UA-mediated inflammatory and oxidative changes in adipocytes [44]. It was shown that mature adipocytes produce and secrete UA [45], and that obesity is associated with increased gene expression of XOR and UA secretion [46]. Moreover, higher waist circumference accompanied by higher leptin production was previously associated with high UA levels [47,48]. An alternative hypothesis assumes that hyperinsulinemia is often associated with hypertriglyceridemia, which is linked to the synthesis of fatty acids in the liver and also to increased de novo purine synthesis, thus giving rise to increased UA levels [49,50]. UA also inhibits AMP-activated protein kinase [51] and stimulates hepatic lipogenesis [52]. Strong positive correlations between UA levels and pre-gestational BMI, and also higher UA levels among GDM women with the occurrence of pre-gestational obesity in our study are, thus, not surprising, because obesity and metabolic syndrome are associated with low-grade chronic inflammation, and increased UA levels could act as a protection against moderate oxidative stress resulting from this situation.

Similarly to others [22,25], we did not find increased UA levels during mid-trimester or postpartum in GDM women compared to controls. Among GDM women, UA levels were positively correlated with pre-gestational BMI, and respective glycemia levels in oGTT and elevated UA levels were also found in women with pre-gestational overweight/obesity, those who required insulin therapy, and women with increased risk for adverse perinatal outcomes. The DIP group and also the group of participants with postpartum glucose metabolism disturbance were unfortunately small, thus impacting on statistical power to detect significant differences in X and UA levels. Therefore, despite a clear association of UA levels with the high-risk GDM subtype regarding the perinatal outcomes, the predictive potential of UA for permanent diabetes development in post-GDM women still remains a question. Molęda et al. [53] reported a possible link between disturbances in carbohydrate metabolism in post-GDM women which were highly correlated with increased UA levels. Similarly to our research, Molęda et al. [53] described a connection between higher UA levels and overweight, obesity, and severity of carbohydrate abnormalities, as well as a positive correlation with respective parameters of oGTT; however, this study did not contain data about UA in an early postpartum period.

Despite the fact that increased insulin resistance (physiologically occurring in the mid-trimester) is positively associated with UA levels even in non-pregnant subjects [54], our results show that X and UA levels raised postpartum in both groups. Lower mid-gestation UA levels are most likely mediated by pregnancy-induced hypervolemia and gestational changes in the renal system (highest increase in extracellular fluid volume, renal blood flow, and glomerular filtration rate coinciding with the second trimester) [55]. Lower UA levels during pregnancy can be also related to higher rates of oxidative stress [56] and subsequent UA consumption as a potent antioxidant. Studied perinatal outcomes of the GDM and non-GDM groups were comparable except for the rate of caesarean section, which was higher in GDM group. No associations of mid-gestational UA levels with pathological values of perinatal outcomes were found; only postpartum levels of X and UA were negatively correlated with gestational age at delivery, and postpartum X levels were significantly higher in a group of women who underwent caesarean section. It should be noted that we registered only several specific neonatal complications; thus, neonatal morbidity may have been higher in total.

In genome-wide association studies, more than 30 loci associated with UA levels were identified, so far explaining 7% of UA variability between individuals [9]. For example, different polymorphisms were identified in the *SLC2A9* gene (GLUT9) encoding a glucose and UA transporter [11,57]. Moreover, rs1014290 in this gene was already associated with decreased T2DM risk in women [58]. In our study, there were no differences in genotype or allele frequencies between GDM women and controls in any of the SNPs analyzed. However, UA levels differed between carriers of genotypes in rs1014290, rs12498742, rs16890979, and rs734553 in GLUT9 postpartum; the statistical significance during pregnancy was lower than 10% in rs1014290, rs12498742, and rs16890979, and could be considered as borderline significant. Four different SNPs in GLUT9 were associated with lower UA levels (allele G in rs1014290 and rs12498742, allele T in rs16890979, and genotype GG in rs734553).

Compared to UA, little is known about X levels in human plasma and the effect of XOR activity on them. We described slightly higher X levels in GDM women during pregnancy and postpartum that may point to increased XOR activity, but this result has to be evaluated. The pro-oxidant and pro-inflammatory actions attributed to UA could be a result of the conversion of X dehydrogenase to X oxidase and the consequent accumulation of reactive oxygen species [1]; thus, the role of X requires further studies. There are certain limitations of current study, such as a relatively small size of the control group (probably explaining the disturbance in Hardy–Weinberg equilibrium) and a lack of participants with physiological pregnancy who passed voluntary postpartum oGTT. Furthermore, the one-year-long postpartum follow-up of post-GDM women was probably too short period to observe the conversion into a permanent type of diabetes, as documented by several studies [16,17].

## 4. Materials and Methods

### 4.1. Characteristics of Study Subjects

The case-control study comprised a total of 143 pregnant Caucasian women enrolled from a defined geographical area (South Moravian district of Czech Republic). The study was performed in accordance with the principles of the Declaration of Helsinki and was approved by the Ethical Committee of Faculty of Medicine, Masaryk University Brno (approval number 22/2010, date of approval 16 September 2010). Informed consent was obtained from all subjects prior to their inclusion in the study. Exclusion criteria were type 1 diabetes, latent autoimmune diabetes of adults (LADA), maturity-onset diabetes of the young (MODY), or T2DM before pregnancy, non-Caucasian origin, hypertensive disorders prior to pregnancy, multiple pregnancies, and severe comorbidities. Controls (*n* = 34) were women with physiological pregnancies who passed routine GDM screening in mid-trimester with negative results. GDM cases (*n* = 109) were women that fulfilled GDM criteria in mid-gestational screening and who also participated in postpartum oGTT (within the period of six weeks to 12 months after delivery). All women underwent mandatory oGTT between the 24th and 28th weeks of pregnancy. Mid-gestational GDM screening was carried out using oGTT with 75 g of glucose load between the 24th and 28th weeks of pregnancy with the following thresholds (modified World Health Organization (WHO) criteria at the time of enrolment in 2012–2013): FPG > 5.6 mmol/L, 1-h PPG > 8.9 mmol/L, and 2-h PPG > 7.8 mmol/L. GDM women were followed from the time of GDM diagnosis until giving birth at the Diabetes Centre of the University Hospital Brno. The treatment for GDM included diet in all cases; 37.8% of GDM cases required insulin therapy. Glucose responses during oGTT were calculated from the area under the curve (AUC) using the trapezoidal rule. Postpartum screening was carried out from six weeks to 12 months after delivery, and diagnosis of diabetes/prediabetes was based on the WHO criteria for non-pregnant subjects: FPG ≥ 7 mmol/L alone or 2-h PPG ≥ 11.1 mmol/L for DM, and FPG 5.6–6.9 mmol/L or 2-h PPG 7.8–11.0 mmol/L for prediabetes.

### 4.2. Blood Sampling, and Uric Acid and Xanthine Measurement

Samples of peripheral ethylenediaminetetraacetic acid (EDTA)/blood were taken from each study participant during their scheduled visit to the prenatal or diabetes center at mid-trimester (between the 24th and 28th weeks of pregnancy) and repeatedly from six weeks to 12 months postpartum in the post-GDM group only. Plasma was separated by centrifugation (1000× *g*, 10 min, 4 °C) and stored at −75 °C until analysis. Plasma UA and X levels were determined using reversed-phase HPLC with ultraviolet detector (HPLC-UV) with a photo-diode array detector, as previously published [59].

### 4.3. Genetic Analysis

The phenol–chloroform method was used for DNA extraction from peripheral blood leukocytes. Five SNPs in genes for UA transporters *ABCG2* and *SLC2A9* (encoding GLUT9) were selected based on their reported significance for UA transport. SNPs were genotyped by polymerase chain reaction with fluorescent-based chemistry (TaqMan^®^ SNP Genotyping Assay, Applied Biosystems, California, USA) according to the manufacturer’s instructions. Genotyping was performed using ABI Prism 7000 (Thermo Fisher Scientific, Waltham, MA, USA). Details on studied SNPs are provided in Table 3.

### 4.4. Statistics

Data were expressed as medians and interquartile ranges (IQR) or proportions. Nonparametric tests were used for comparison between and within the groups (Mann–Whitney test, Fisher exact test, Kruskal–Wallis ANOVA, and Wilcoxon test). Correlations were computed using Spearman correlation coefficients. Chi-square test was used for contingency tables. The Statistica software (StatSoft, Tulsa, OK, USA) was used for all analyses. A *p*-value <0.05 was considered as statistically significant.

## 5. Conclusions

Our data suggest an association of UA levels with impaired glucose tolerance within GDM women during pregnancy. The correlations of UA levels among GDM women with respective glycemia levels in oGTT show a link between UA levels and impaired glucose metabolism during pregnancy. Furthermore, the positive correlation of pre-gestational BMI with UA levels and GDM diagnosis provides the possible connection among overweight/obesity, low-grade inflammation, hyperuricemia, and impaired glucose tolerance. Higher levels of UA in a sub-group of GDM patients treated by insulin in pregnancy and within the risk group for adverse perinatal outcomes point to possible UA metabolism disturbance associated with a more severe glucose homeostasis problem. We described genotype–phenotype associations between SNPs in UA transporter GLUT9 (encoded by the *SLC2A9* gene) and UA levels. The putative effect of UA levels on permanent type diabetes risk requires further study because only 5.9% of women developed a persistent form of DM within the first year after delivery.

## Figures and Tables

**Table 1:** Clinical and anthropometric characteristics of study subjects.

Parameter	GDM (*n* = 109)	Controls (*n* = 34)	*p*
Age (years)	33 (30–35)	31 (28–33)	0.040
Pre-gestational BMI (kg m^-2^)	24.6 (21.2–28.7)	22.5 (20.6–27.0)	NS
Weight gain during pregnancy (kg)	8 (5–10)	13 (10–17)	<1 × 10^−6^
Offspring birth weight (g)	3120 (2790–3500)	3355 (3070–3700)	0.032
Primiparity	38.5%	44.1%	NS
History of previous GDM	26.2%	0%	0.009
Family history of DM	72.1%	27.3%	<1 × 10^−4^
FPG (mmol/L) mid-gestation	4.8 (4.4–5.2)	4.2 (4.0–4.4)	<1 × 10^−6^
1-h PPG (mmol/L) mid-gestation	9.4 (8.9–10.1)	5.9 (5.3–6.5)	<1 × 10^−6^
2-h PPG (mmol/L) mid-gestation	8.2 (7.7–8.8)	5.5 (4.8–6.1)	<1 × 10^−6^
AUC (mmol/L/h) mid-gestation	13.0 (12.3–13.7)	9.6 (8.9–10.3)	<1 × 10^−6^
FPG (mmol/L) postpartum	4.7 (4.4–4.9)	-	-
1-h PPG (mmol/L) postpartum	6.3 (5.1–7.8)	-	-
2-h PPG (mmol L^-1^) postpartum	5.0 (4.3–5.9)	-	-
AUC (mmol/L/h) postpartum	9.7 (8.8–10.6)	-	-
X levels (μmol/L) mid-gestation	3.61 (3.41–3.85)	3.41 (3.21–3.70)	0.049
X levels (μmol/L) postpartum	4.14 (3.91–4.61)	3.79 (3.53–4.20)	0.0002
UA levels (μmol/L) mid-gestation	183 (154–205)	178 (168–190)	NS
UA levels (μmol/L) postpartum	221(195–249)	213 (190–248)	NS

Data expressed as medians and interquartile ranges (IQR) or proportions (%). Differences were evaluated by nonparametric Mann-Whitney or Fisher exact tests. 1-h PPG—1-h post-load plasma glucose; 2-h PPG—2-h post-load plasma glucose; AUC—area under the curve of oral glucose tolerance test calculated using trapezoidal rule; DM—diabetes mellitus; FPG—fasting plasma glucose; GDM—gestational diabetes mellitus; NS—not significant; UA—uric acid; X—xanthine; - means missing data.

**Table 2:** Labor and perinatal data of study subjects.

Parameter	GDM (*n* = 57)	Controls (*n* = 34)	*p*
Primiparity	57.9%	44.1%	NS
Gestational age at delivery (weeks)	40 (39–40)	40 (39–40.5)	NS
Premature labor	12.3%	0%	NS
Spontaneous onset of labor	54.4%	66.0%	NS
Protracted labor (>480 min)	3.51%	4.55%	NS
Caesarean section rate	33.3%	13.6%	0.043
Complications following childbirth	5.26%	4.55%	NS
Pathological Apgar score	5.26%	0%	NS
Pathological umbilical cord-blood pH	1.75%	4.55%	NS
Pathological umbilical cord base excess	3.51%	0%	NS

Data are expressed as medians and interquartile ranges (IQR) or proportions (%). Differences were evaluated by nonparametric Mann–Whitney or Fisher exact tests. GDM—gestational diabetes mellitus; NS—not significant.

**Table 3:** Selected single-nucleotide polymorphisms (SNPs) within genes encoding uric acid transporters.

UA transporter (*gene*)	SNP	Nucleotide Substitution	SNP Effect (Position)	MAF in GDM Group (%)	MAF in Control Group (%)	*p* (Genotype and Allele Frequency)
ABCG2 (*ABCG2*)	rs2231142	G/T	141 Q/K (exon)	T 10.5	T 4.69	both NS
GLUT9 (*SLC2A9*)	rs1014290	A/G	(intron)	G 35.6	G 29.3	both NS
	rs12498742	A/G	(intron)	G 29.5	G 37.9 *	both NS
	rs16890979	C/T	253 V/I (exon)	T 26.6	T 47.9	both NS
	rs734553	G/T	(intron)	G 28.0	G29.0	both NS

Chi-square test was used for differences in genotype and allele frequencies. ABCG2 transporter and *ABCG*—ATP binding cassette subfamily G member 2; GDM—gestational diabetes mellitus; GLUT9—glucose transporter 9; MAF—minor allele frequency; NS—not significant; *SLC2A9*—solute carrier family 2 member 9; UA—uric acid. * This population is not in Hardy–Weinberg equilibrium.

## Abbreviations

1-h PPG	1-h post-load plasma glucose
2-h PPG	2-h post-load plasma glucose
ABCG2	ATP-binding cassette subfamily G member 2
AMP	adenosine monophosphate
ANOVA	analysis of variance
AUC	area under the curve
BMI	body mass index
DIP	diabetes in pregnancy
DM	diabetes mellitus
DNA	deoxyribonucleic acid
EDTA	ethylenediaminetetraacetic acid
FPG	fasting plasma glucose
GDM	gestational diabetes mellitus
GLUT9	glucose transporter 9
HOMA-IR	homeostatic model assessment for insulin resistance
HPLC-UV	high-performance liquid chromatography with ultraviolet detector
IQR	interquartile ranges
LADA	latent autoimmune diabetes of adults
MAF	minor allele frequency
MODY	maturity-onset diabetes of the young
NS	not significant
oGTT	oral glucose tolerance test
*p*	probability
SLC2A9	solute carrier family 2 member 9
SNPs	single-nucleotide polymorphisms
T2DM	type 2 diabetes mellitus
UA	uric acid
WHO	World Health Organization
X	xanthine
XOR	xanthine oxidoreductase
